# Different Modes of Transactivation of Bacteriophage Mu Late Promoters by Transcription Factor C

**DOI:** 10.1371/journal.pone.0129504

**Published:** 2015-06-09

**Authors:** Ganduri Swapna, Vandana Kumari, Valakunja Nagaraja

**Affiliations:** 1 Department of Microbiology and Cell Biology, Indian Institute of Science, Bangalore, India; 2 Jawaharlal Nehru Centre for Advanced Scientific Research, Bangalore, India; University of Helsinki, FINLAND

## Abstract

Transactivator protein C is required for the expression of bacteriophage Mu late genes from *lys*, *I*, *P* and *mom* promoters during lytic life cycle of the phage. The mechanism of transcription activation of *mom* gene by C protein is well understood. C activates transcription at P*mom* by initial unwinding of the promoter DNA, thereby facilitating RNA polymerase (RNAP) recruitment. Subsequently, C interacts with the ß' subunit of RNAP to enhance promoter clearance. The mechanism by which C activates other late genes of the phage is not known. We carried out promoter-polymerase interaction studies with all the late gene promoters to determine the individual step of C mediated activation. Unlike at P*_mom_*, at the other three promoters, RNAP recruitment and closed complex formation are not C dependent. Instead, the action of C at P*_lys_*, P*_I_*, and P*_P_* is during the isomerization from closed complex to open complex with no apparent effect at other steps of initiation pathway. The mechanism of transcription activation of *mom* and other late promoters by their common activator is different. This distinction in the mode of activation (promoter recruitment and escape versus isomerization) by the same activator at different promoters appears to be important for optimized expression of each of the late genes.

## Introduction

**Fig 1:**
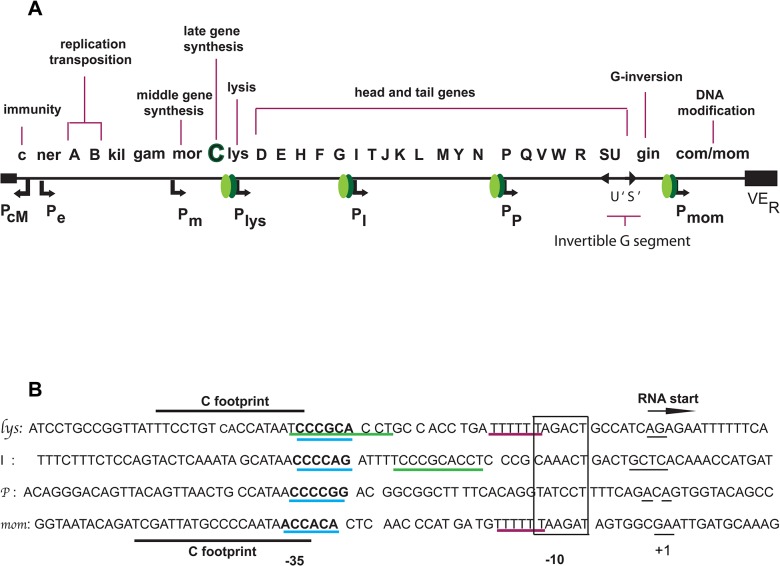
Phage Mu late genes. **A.** Linear genome organization of phage Mu. The middle gene product C (shown in Green and represented as dimer) activates transcription from the late gene promoters *lys*, *I*, *P* and *mom*. **B.** Mu late promoter sequences. Late promoter sequences were aligned with respect to their transcription start sites as determined by S1 nuclease mapping (Bolker, *et al*., 1989, Margolin, *et al*., 1989), and similarities in -10 and -35 regions. Heavy black line indicates the C-foot print in *lys* and *mom* promoters. A 10bp match between *lys* and *I* promoters and the 6T stretch common between *lys* and *mom* promoters in the spacer region are underlined in green and magenta respectively. Sequences of non-consensus -10 (boxed) and -35 elements (underlined in blue) are depicted.

Bacteriophage Mu utilizes the host RNA polymerase for its transcription. Expression of the genes in phage Mu is regulated in a temporal fashion. Like in other phages, the Mu genome is divided into transcription units designated as early, middle and late genes based on their timing of expression during the phage life cycle. Mu late genes that are expressed during the last phase of the lytic cycle, subsequent to the initiation of the phage DNA replication are dependent on C protein for their expression (Fig 1) [1]. Among the four late genes, the *lys* gene product is required for host cell lysis at the end of the cycle. Mutants defective in *lys* make normal amounts of functional phage particles but do not release them from the cell [2,3]. *I* gene has been shown to be required for head synthesis and possibly involved in protein scaffolding during head assembly [3,4], while *P* gene encodes for one of the enzymes involved in the synthesis of phage tail [3,4]. The *mom* gene encodes for a unique DNA modification function [5,6] that confers an anti-restriction phenotype to the phage genome making it refractile to the host restriction endonucleases [7]. Although *mom* is not essential, it confers survival advantage to the genome [7]. Notably, its untimely or inappropriate high level expression leads to cell death owing to its unique DNA modification function [8,9].

The unusual DNA modification by Mom and the associated cytotoxicity due to its expression formed the basis for the detailed analysis of its regulation. A variety of regulatory measures operational at transcriptional and post-transcriptional levels are well elucidated, including, C mediated transactivation [8,10–14]. C binds at its site overlapping the *mom* promoter, alters the DNA conformation by unwinding, thereby, facilitating RNAP recruitment at the otherwise out-of phase promoter elements unmalleable for enzyme occupancy [15,16]. In the next step, C interacts with ß' subunit of RNAP, inducing allosteric transitions in the enzyme active site and thus enhancing promoter clearance [17,18]. However, little is known about the mechanism of activator mediated transactivation at *lys*, *I*, *P* promoters which are also expressed only in late phase of lytic cycle in C dependent manner [1,14,19].

In the present study, we set out to determine the facets of C mediated transactivation at *lys*, *I*, *P* promoters, asking the following questions: Would the mechanism at these promoters involve a multi-step control as seen at P_*mom*_? Is C binding a prerequisite for RNAP occupancy at these promoters? Alternatively, does it follow a single step mechanism typically seen with a majority of promoters subjected to activation? We describe the promoter-polymerase interaction studies in presence of C, aimed to understand C function at *lys*, *I* and *P* promoters. We demonstrate that the mode of action by C at these promoters is distinct to the mechanism seen at P*mom*.

## Results

### Recruitment of RNAP at the *lys*, *I* and *P* promoters does not require C protein

**Fig 2:**
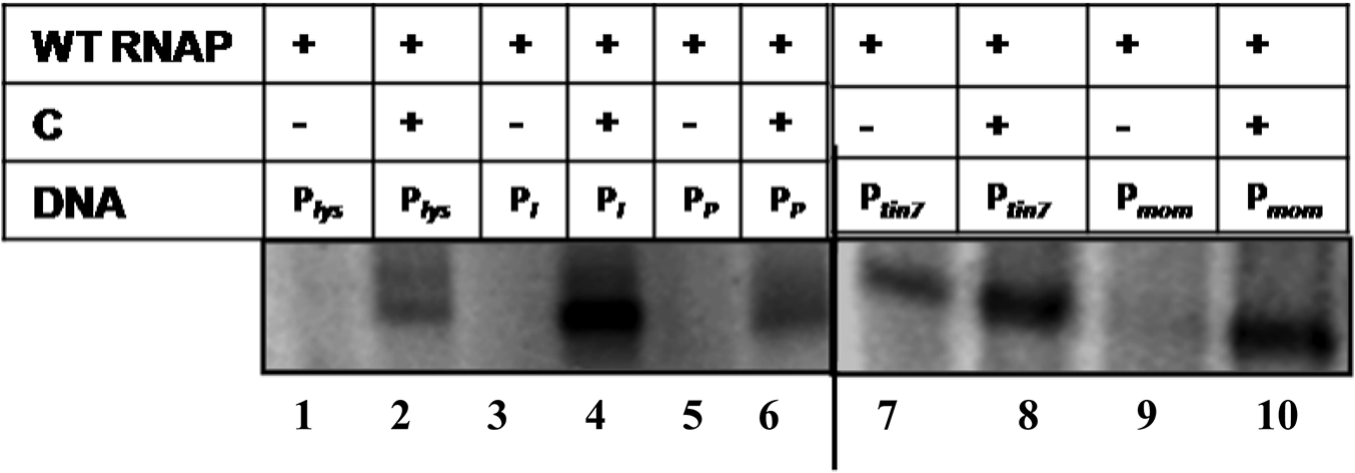
C is essential for transactivation from late promoters. *In vitro* transcription assays carried out on *lys*,*I*,*P* (lanes 1–6), and at P_*mom*_ (lanes 9–10) promoters in the absence and presence of transactivator C. At mutant *mom* promoter—P_*tin7*,_ RNAP recruitment is C independent. The basal transcription in the absence of C is enhanced in its presence due to second step activation described in the text (lanes 7–8).

**Fig 3:**
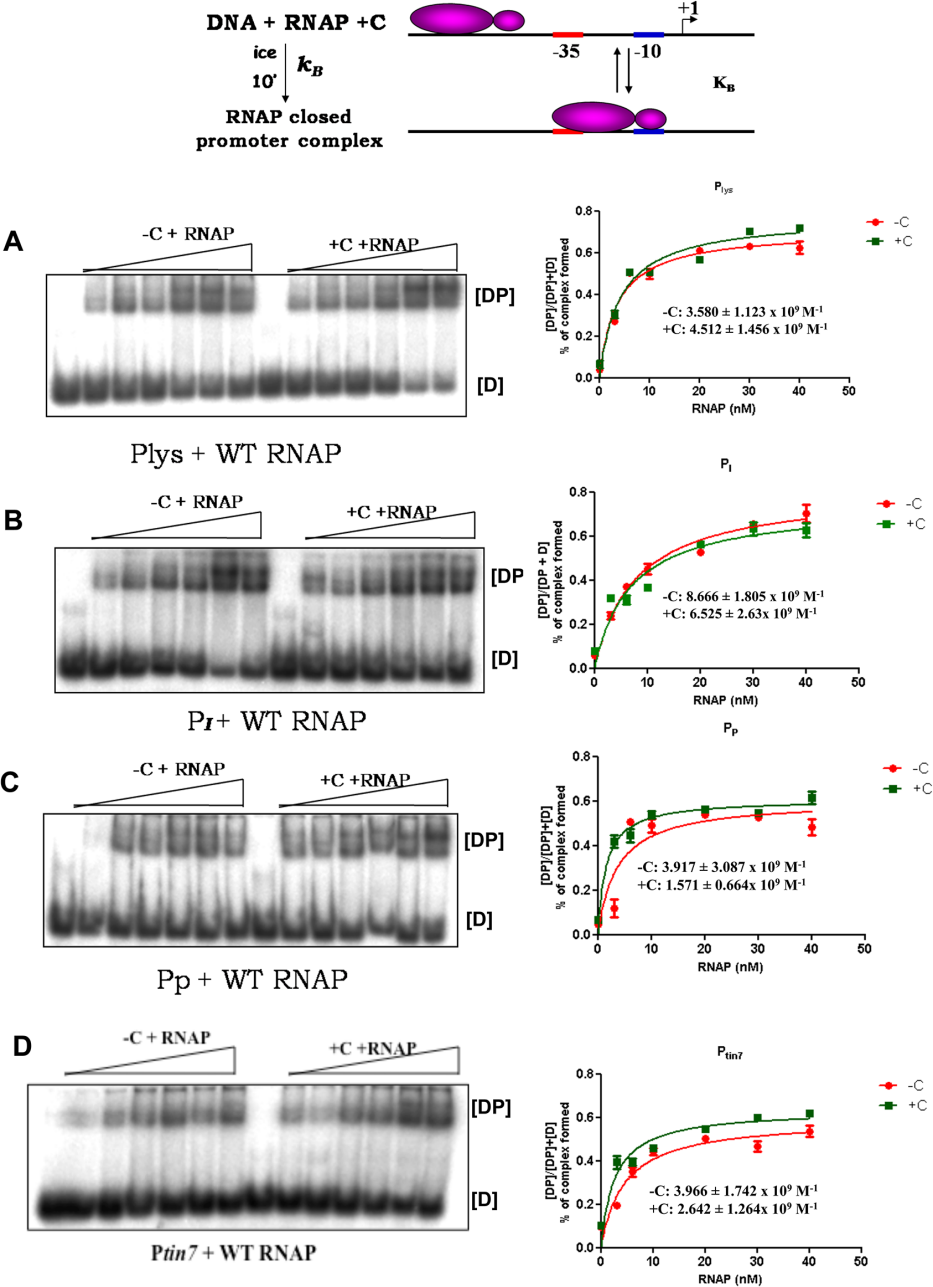
C protein is not essential for recruitment of RNAP at *lys*, *I* and *P* promoters. 5′ P^32^ labeled *lys*, *I*, *P*, *tin7* promoter constructs were incubated with increasing concentration of RNAP either in the absence or presence of C protein on ice for 10 min and samples were analyzed on 3.5% native PAGE at 4°C. The amount of free DNA [D] and RNAP-bound promoter DNA [DP] were quantified to determine the K_*B*_ of RNAP binding. DP:D values were plotted as a function of RNAP concentration. **A-D** represent- the promoter binding affinity of RNAP in the absence and presence of C protein at *lys*, *I*, *P and tin7* promoters respectively. RNAP binds these promoters irrespective of the presence of C protein. The results are representative of three independent experiments.

*In vitro* transcription analysis carried out on the templates generated from the late promoter clones revealed that C is essential for transactivation from the *lys*, *I* and *P* promoters similar to that at P_*mom*_. No specific transcription was observed at these promoters in the absence of the C protein (Fig 2), confirming their dependence on C described earlier [14,19]. To understand the mechanism of activation by C at these promoters, individual steps of transcription initiation pathway were assessed. To address whether there is any requirement of transactivator C for the recruitment of RNAP at the *lys*, *I* and *P* promoters, Electrophoretic Mobilist Shift Assay (EMSA) was carried out on the end-labeled late promoter DNA fragments. DNA binding (*K*_*B*_) was estimated by measuring the intensity of DNA-protein complex and plotting the value as a function of RNAP concentration (Fig 3A–3C). Similar experiments were carried out on transactivator independent *mom* promoter mutant-P*tin7* as a control, where RNAP recruitment occurs in the absence of C protein (Fig 3D). From the data, it is evident that C does not have an effect at the step of closed complex formation, as the K_*B*_ values in the presence or absence of C were comparable. Thus, unlike at P_*mom*_ where C facilitates RNAP recruitment by unwinding the DNA [15,16], RNAP recruitment appears to be C independent at *lys*, *I* and *P* promoters. Moreover, the affinity of RNAP to these late promoters is comparable and not altered in presence of C. These results are markedly contrasting to RNAP binding at P_*mom*_ [17,20,21].

### Influence of C protein at the step of isomerization and transcription elongation

**Fig 4:**
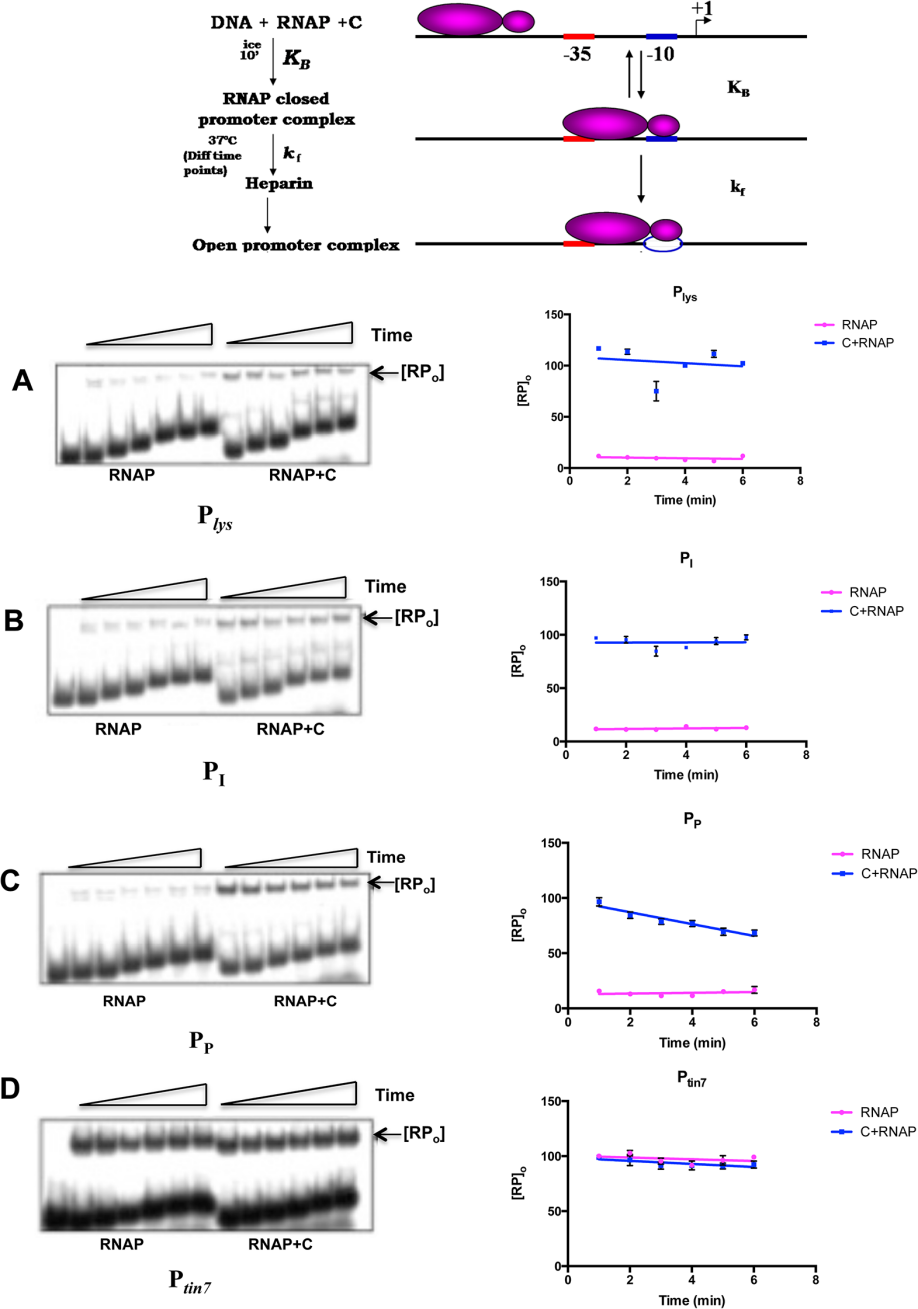
C enhances the isomerization of closed complex to promoter open complex. Open complex formation assays were carried out on 5′ end labeled promoter constructs as described in Experimental procedures. C was added to the reaction wherever indicated. RNAP-promoter open complexes [RP_*O*_] were resolved on 3.5% native page. **A-C** indicate the requirement of C protein for effective open complex formation at *lys*,*I*,*P* promoters respectively. **D.** RPo formation on *tin7* promoter is C- independent. The open complexes were quantified using Multi gauge software. The complex formed at each promoter with RNAP in presence of C was taken as 100% and values were normalized accordingly. The results are an average of three independent experiments.

Conversion of closed complex to open complex is the next step during initiation and also one of the major steps for transcriptional regulation at a number of promoters. Many transactivator proteins exert their effect at this stage [22–26]. Previous studies with P_*tin7*_, a mutant *mom* promoter in which C is not required for RNAP binding [20], revealed that C does not enhance the formation of open complex at this promoter [17]. Similar experiments were carried out to study the effect of C on the open complex formation at *lys*, *I* and *P* promoters as described in Materials and Methods. In all the three promoters, C facilitated open complex formation. Although very faint heparin resistant complexes were observed in the absence of C (see later section), they are likely to be non-functional open complexes (see later section/paragraph). As shown earlier, extent of formation of open complex was unaltered in the presence and absence of C at P_*tin7*_ compared to that observed at *lys*, *I* and *P* promoters (Fig 4A–4D).

**Fig 5:**
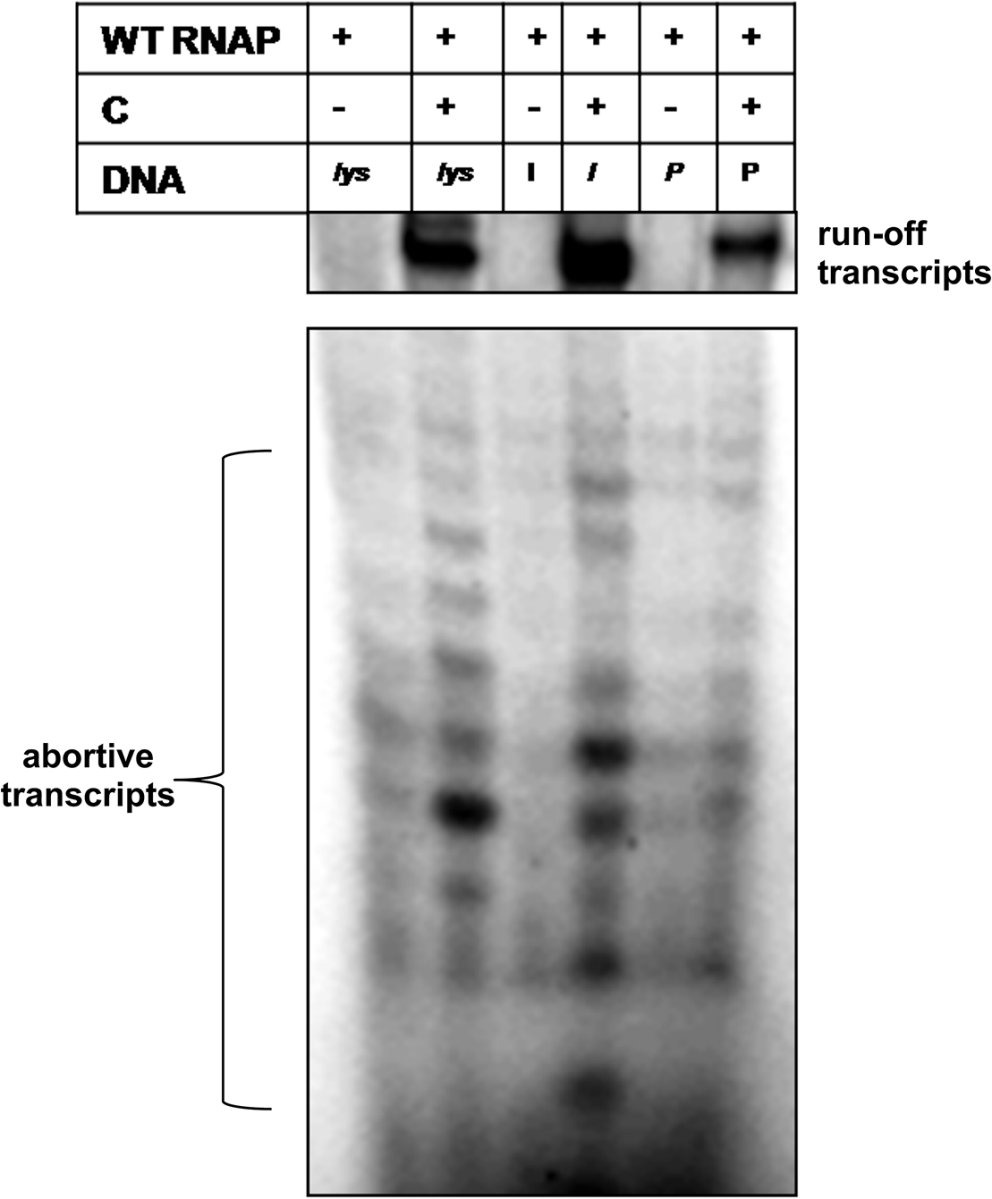
Transactivator C facilitates productive transcription at *lys*, *I*, *P* promoters. Abortive transcription profile of *lys*, *I*, *P* promoters. Low amounts of abortive transcripts are seen at *lys*, *I*, *P* in the absence of C protein but no productive transcription is observed. In the presence of C, though the abortive transcription is seen, RNAP enters into productive elongation phase and transcripts are synthesized.

At P_*mom*_ (and also at P_*tin7*_), C enhances promoter escape by overcoming abortive initiation [17]. To address the effect of C during promoter clearance step of transcription initiation at *lys*, *I*, and *P* promoters, *in vitro* transcription assays were carried out. The abortive initiation profiles at each of these promoters were compared in the presence or absence of C in the reaction. C is required for the RNAP to enter a productive elongation phase at *lys*, *I*, *P* promoters (Fig 5). The presence of lower levels of abortive transcripts at these promoters (Fig 5 lanes1, 3, 5) could be attributed to the weak complexes formed in the absence of C protein (Fig 4A–4C). These open complexes appear to synthesize only the abortive transcripts that fail to enter productive elongation phase in the absence of transactivator C (Fig 5 lanes 1, 3, 5). These results suggest the formation of a moribund complex/dead-end complex [27] by RNAP at *lys*, *I*, *P* promoters in the absence of transactivator C. Thus, at these three promoters, C is required for productive open complex formation leading to efficient promoter escape and transcription elongation.

### Effect of G524D*rpoC* RNAP on transactivation from *lys*, *I*, *P* promoters

**Fig 6:**
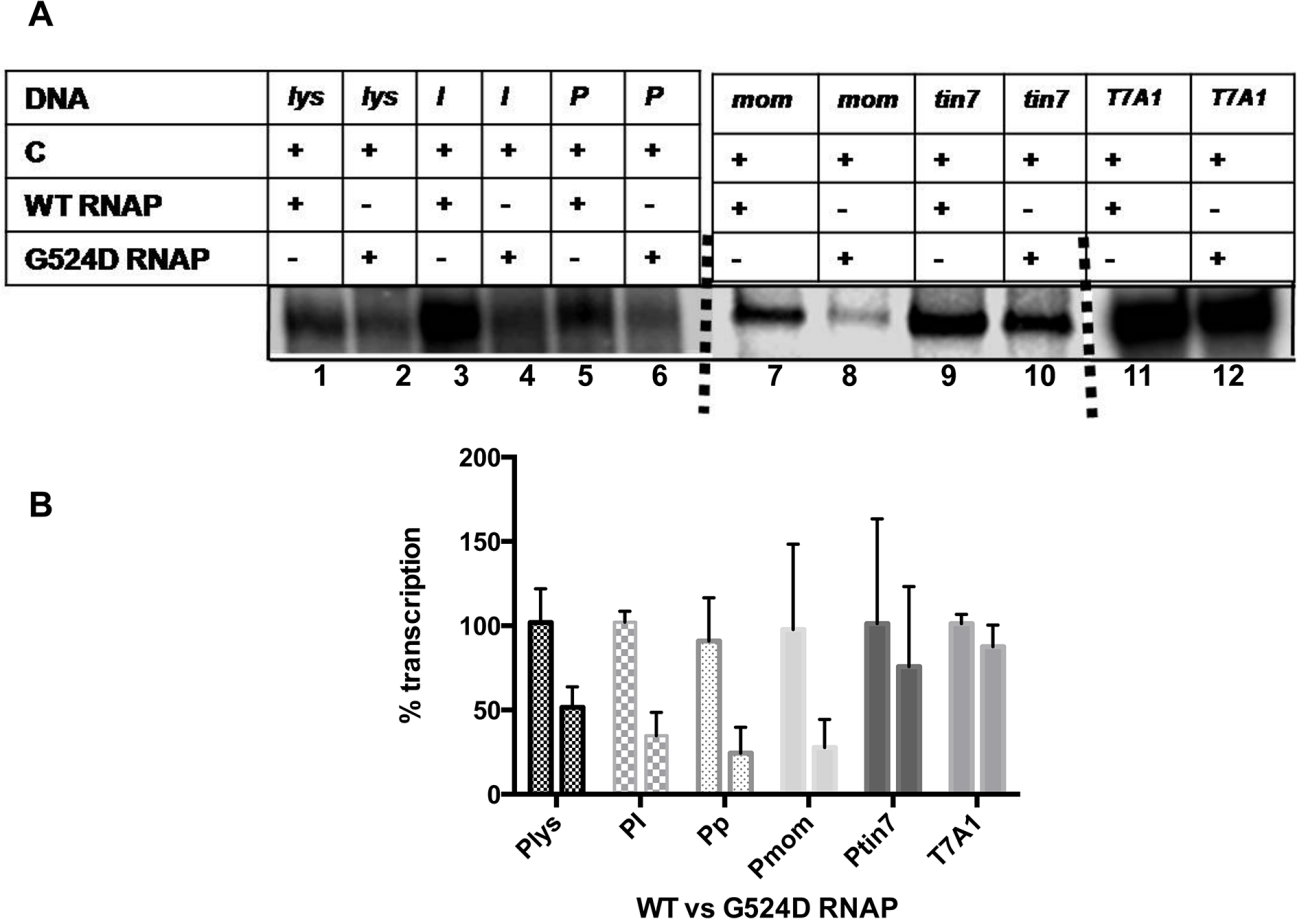
G524D*rpoC*RNAP exhibits defective transcription from other late promoters similar to that observed at P_*mom*_. *In vitro* transcription assays were carried out on phage Mu late promoter constructs (*lys*,*I*,*P*, *mom* and mutant *mom* promoter-*tin7*) and *E*. *coli* ^70^ promoter *T7A1*. A) Samples were analyzed on 8% denaturing PAGE to assess the productive transcripts and quantified using Muti gauge software. Transcription at each individual promoter with WT RNAP was taken as 100% and the values were normalized. B) Bar diagram depicts quantitative representation of transcription efficiency. G524D RNAP showed reduced productive transcription at all C- dependent promoters compared to WT RNAP but was competent at *T7A1* promoter. The results are an average of three independent experiments.

The interaction between DNA bound transactivator C and the β′ subunit of RNAP, and the concomitant allosteric changes in the enzyme have been shown to be important for RNAP to enter into productive elongation phase at P_*mom*_ [18]. Positive control (*pc*) mutants of C or specific mutations in ß' subunit of RNAP affected this second step transactivation at P_*mom*_ [17,18]. P_*mom*_ specific transactivation deficient mutant of *rpoC* (ß' subunit) G524D*rpoC* RNAP [18] isolated in our previous studies was used to understand the effect of the mutation on transcription at the late gene promoters. Single round transcriptions were carried out from *lys*, *I*, *P*, *mom* and *tin7* promoters with wild type (WT) and G524D RNAP. At P_*mom*_ and P_*tin7*_ where C exerts a dual step activation mechanism, G524D RNAP exhibited reduced transcription in the presence of C (Fig 6A, lanes 7–10) [18]. The same effect was observed with the mutant RNAP at *lys*, *I* and *P* promoters, although at these promoters effect of C mediated activation is only at isomerization step during promoter polymerase interaction (Fig 6A, lanes 1–6). Notably, WT and mutant RNAP transcribe with equal efficiency from *T7A1* promoter, which is not subjected to C control (Fig 6A, lanes 11, 12). Transcription efficiencies of WT and G524D RNAP at late promoters is quantitated and represented as bar diagram (Fig 6B). RNAP ß' subunit interaction with C, thus seems to be critical for transcription initiation from all the phage Mu late genes.

## Discussion

The mechanism of C-mediated activation from P_*lys*_, P_*I*_ and P_*P*_ appears to be significantly different from P_*mom*_. C is not required for RNAP recruitment *per se* at these promoters unlike at P_*mom*_, where RNAP cannot bind the promoter in the absence of C [17,20]. At these promoters, C exerts an effect at the step of open complex formation, not seen with P_*mom*_. Faint open complexes observed with these promoters in the absence of C (Fig 4A–4C), seem to be transcriptionally inactive as they are not converted into productive transcripts (Fig 2 and Fig 5). The abortive transcription seen at these promoters in the absence of C may be a consequence of formation of the ternary complexes termed moribund complexes described earlier at other promoters, which do not proceed into a productive elongation phase [27,28].

**Fig 7:**
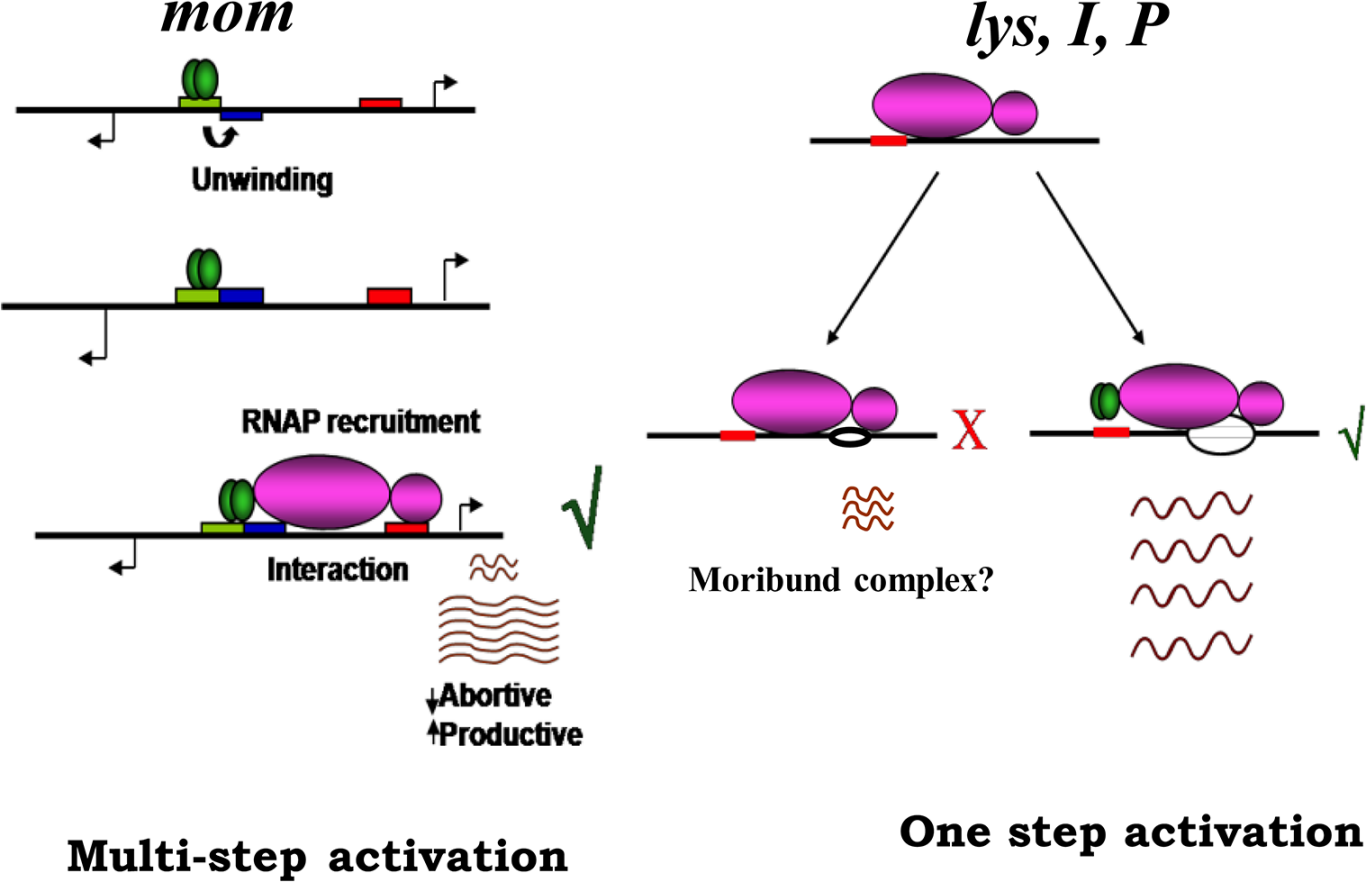
Differential activation by C at late promoters. Cartoonic representation of the mechanism of C mediated transactivation at late promoters. C activates transcription by a multi-step mechanism at P_*mom*._ C acts initially to recruit RNAP to the mom promoter and then facilitates promoter escape. At promoters *lys*, *I* and *P-* C acts at a single step and facilitates open complex formation which proceeds into productive elongation mode. In the absence of activator C, the open complex formed remains transcriptionally inactive. RNAP holoenzyme—magenta, C dimer-green oval, out of phase promoter elements at P_*mom*_ is represented on DNA as blue and red rectangles respectively. CBS (C-binding site) over lapping -35 element is shown as green rectangle. CBS on P_*lys*_,P_*I*_,P_*P*_ promoters is depicted as red rectangle. RNAP open complex at *lys*, *I*, *P* promoters is shown as open circle.

How these results compare to the activation mechanisms studied earlier? Some of the well studied activators exert different roles at different promoters. CAP, a global regulator of gene expression in prokaryotes, activates transcription from a number of promoters exerting different modes of activation based on the location of its binding site on the DNA and its interaction surface on RNAP [26]. By interacting with α-CTD of RNAP, CAP activates transcription from the *lac* promoter by enhancing the closed complex formation. However, at the *galP1* promoter, it binds with α-NTD and enhances the isomerization of the closed complex to a transcriptionally competent open complex [24,26]. λcI activates its own expression from the λ*P*_*RM*_ promoter by enhancing the rate of isomerization, *K*_*f*_ [29], by contacting the σ-CTD of RNAP [23,26,30]. Simultaneously, it represses transcription from the divergently positioned λ*P*_*R*_ promoter. λcI also stimulates closed complex formation (K_*B*_) at the P_*RM*_ promoter with a mutant polymerase Eσ^70^—R596H RNAP, but not in presence of WT RNAP [31]. The GalR protein that regulates the *gal* operon in *E*. *coli* is also shown to exhibit differential effect at the promoters *P1* and *P2*. Its binding to the DNA on the same face of *P1* represses *P1* transcription, while stimulating transcription from *P2* located on the opposite face of DNA [32]. The p4 protein of bacteriophage ϕ29 represses transcription from the early promoter *A2c*, but activates late gene promoter *A3* [33,34], by interacting with the α subunit of RNAP [33–35]. The activator facilitates RNAP recruitment at the *A2c* promoter but prevents the elongation step by inhibiting promoter clearance [34]. Activation of transcription from A3 promoter by the protein is achieved through stabilization of closed complex [36]. In a majority of these examples, the regulatory protein binding site is at varied distance with respect to promoter location. However, at phage Mu late promoters, the activator binding site is positioned at upstream of the -35 site (Fig 1B). Normally, activators bound next to -35 elements influence the initiation process by contacting α-NTD or σ subunits. However, C does not interact with either of these subunits [18]. In our previous studies, we demonstrated that C interacts with ß' subunit to induce allosteric transitions that facilitated the promoter escape at P_*mom*_ [18]. The data presented with the mutant RNAP in Fig 6 suggest the importance interaction of C with the ß' subunit at the other three late promoters. Thus, although there are differences in the mechanism of C mediated transactivation between P_*mom*_ and other late promoters, similarities are seen in RNAP interaction**.** Studies with G524D RNAP showed defective transcription with all the four late promoters, indicating the importance of functional interaction between ß' subunit and C to facilitate transactivation. On one hand, the functional interaction is needed for promoter escape at second step of activation of P_*mom*_ while with other promoters, this interaction appears to be required for isomerization step (Fig 7).

Why a given activator would function in a mechanistically different fashion at different promoters? More specifically, how to envision C acting differently at *lys*, *I* and *P* promoters compared to its action at P_*mom*_? Analysis of the late promoter sequences indicates a variation in the architecture of the promoter elements (-10, -35 elements and spacer region) and the differences in C-binding site (Fig 1B). These features might lend to differences in the C interaction at these promoters and also differences in RNAP binding. Further, the effects of an activator at different kinetic steps could depend on when during the initiation process the activator-RNAP interaction occurs [37].

Finally, what is the biological relevance of such a difference in the regulation of the four late promoters? After the switch from middle to late gene transition, the phage has to ensure that the proteins required for morphogenesis are present in sufficient quantities. In contrast, the Mom protein is required in small quantities for a limited DNA modification and its pre-mature or over-expression causes toxicity[8,9]. Thus, elaborate negative regulatory measures and dual step activation of P*mom* is a necessity that is not required for other late gene promoters engaged in production of phage structural proteins.

## Materials and Methods

### Chemicals and Reagents

NTPs and dNTPs were purchased from Promega. [γ^32^P] ATP and [α^32^P] UTP were purchased from PerkinElmer life sciences. All column materials used for protein purifications were from GE Health care. Restriction enzymes were from New England Biolabs. The oligonucleotides and other chemicals used were from Sigma-Aldrich. Sequence of various oligonucleotides used in the study is listed in Table 1. 92 base long top and bottom strands comprising the *lys*, *I* and *P* promoter elements with 5′- EcoRI and 3′-BamHI over-hangs respectively, were designed in such a way that 20nt were present downstream of the transcription start site. The oligonucleotides were either end labeled with [γ^32^P] ATP, annealed and used for electrophoretic mobility shift assays or were annealed and used for cloning between EcoRI-BamHI sites in pUC19.

**Table 1:** Oligonucleotides used in the study.

Oligonucleotide	Description	Sequence
*lys* F	92mer *lys* promoter forward primer with 5′ EcoRI overhang	5′aattcatcctgccggttatttcctgtcaccataatcccgcacctgccacctgattttttagactgccatcagagaattttttcagggaagcg 3′
*lys* R	92mer *lys* promoter reverse primer with 5′ BamHI overhang	5′gatccgcttccctgaaaaaattctctgatggcagtctaaaaaatcaggtggcaggtgcgggattatggtgacaggaaataaccggcaggatg 3′
*I* F	92mer *I* promoter forward primer with 5′ EcoRI overhang	5′aatt**c**tttctttctccagtactcaaatagcataaccccagattttcccgcacctcccgcaaactgactgctcacaaaccatgatgagcagcgg 3′
*I* R	92mer *I* promoter reverse primer with 5′ BamHI overhang	5′gatc**c**cgctgctcatcatggtttgtgagcagtcagtttgcgggaggtgcgggaaaatctggggttatgctatttgagtactggagaaagaaag 3′
*P* F	92mer *P* promoter forward primer with 5′ EcoRI overhang	5′aatt**c**acagggacagttacagttaactgccataaccccggacggcggcttttcacaggtatccttttcagacagtggtacagccacgccccg 3′
*P* R	92mer *P* promoter reverse primer with 5′ BamHI overhang	5′gatccggggcgtggctgtaccactgtctgaaaaggatacctgtgaaaagccgccgtccggggttatggcagttaactgtaactgtccctgtg 3′
*tin7* P2 F	78mer P2 disrupted *tin7* promoter top strand	5′aattccggttgcgccccaataaccacactcaacccatgatgtttgttaagatagtggcgaattgatgcaaaggaggtg 3′
*tin7* P2 R	78mer P2 disrupted *tin7* promoter bottom strand	5′gatccacctcctttgcatcaattcgccactatcttaacaaacatcatgggttgagtgtgtggttattggggcgcaaccggg 3′
T7A1	84mer T7A1 promoter top strand	5′ggatccaatttaaaagagtattgacttaaagtctaacctataggatacttacagccatcgagagggacacggcgaataggaattc 3'
pUC F	17mer forward sequencing primer	5′ gtaaaacgacggccagt 3′
pUC R	17mer reverse sequencing primer	5′ caggaaacagctatgac 3′

### Plasmids, promoter templates and protein purification

The annealed synthetic oligonucleotides with EcoRI-BamHI over-hangs were cloned into pUC19. Transcription templates were generated by PCR amplification of the late promoter plasmids using pUC forward and reverse primers, followed by gel purification. C protein was purified by following the procedure described earlier [38]. WT and mutant RNAPs were purified following Kashlev *et a*l [39], using Ni-NTA sepharose and heparin-sepharose affinity columns.

### RNAP- promoter interaction assays

92-base pair (bp) 5′ P^32^ labeled *lys*, *I*, *P* and 78-bp 5′ P^32^ labeled *tin7* promoter fragments (Table 1) were used in assays for both closed and open complex formation. The assays were carried out either in the presence or absence of C protein, using *E*. *coli* RNAP. The closed complex formation experiments were carried out as described earlier [17,18,40]. Briefly, the promoter fragments were incubated with increasing concentrations of RNAP on ice for 10 min, electrophoresed on a 3.5% native PAGE at 4°C and visualized by phosphorimager. The intensity of the DNA- protein complex formed [DP] and the free DNA [D] was quantified using Multigauge software. DNA binding affinity values of the proteins were determined by taking ratio of the amount of DNA in DNA- protein complex [DP] to that of total DNA {[DP]+[D]} and were plotted as a function of [RNAP] concentration. Open complex formation assays were carried out as described [17], using end labeled promoter fragments and incubating with a fixed concentration of RNAP, in the presence or absence of 2 fold molar excess of C protein. Heparin challenged open complexes were analyzed on 3.5% native PAGE and visualized by phosphorimager. The amount of open complex [RP_O_] formed at these promoters was quantified using Multigauge software.

### *In vitro* transcription assays

Transcription reactions were carried out on the linear DNA templates of P_*lys*_, P_*I*_, P_*P*_, P_*mom*_ and P_*tin7*_ with WT and mutant RNAP in transcription buffer [40 mM Tris-Cl pH 8.0, 5 mM (CH3COO)_2_ Mg, 0.1mM EDTA, 0.1 mM DTT, 100 mM KCl, 100μg/ml BSA]. Reactions were initiated by incubating 40 nM DNA, 80 nM RNAP in transcription buffer on ice for 10 min to allow the formation of closed complex. 300 nM C protein was used wherever required. The reactions were shifted to 37°C for 10 min to allow the formation of open complex. For single round transcriptions, 50μg/ml heparin was added and incubated at 37°C for 1 min. With the addition of 0.3 mM NTP mix and 3μCi [α^32^ P] UTP (6000 Ci/m Mol), the reactions were initiated and after 30 min at 37°C, terminated by the addition of urea loading dye (8M Urea, 0.05% bromophenol blue and 0.05% xylene cyanol), heat inactivated at 65°C for 3 min and quenched on ice. The samples were applied either to a 10% denaturing PAGE or 22% denaturing PAGE for analyzing run- off transcripts and abortive initiation respectively.
